# Osteoarthritis Preoperative Package for care of Orthotics, Rehabilitation, Topical and oral agent Usage and Nutrition to Improve ouTcomes at a Year (OPPORTUNITY); a feasibility study protocol for a randomised controlled trial

**DOI:** 10.1186/s13063-019-3709-5

**Published:** 2020-02-19

**Authors:** A. Hamish R. W. Simpson, Anna Bell-Higgs, Philip G. Conaghan, Peter Craig, David F. Hamilton, Catherine Hankey, Catriona Keerie, Sarah R. Kingsbury, Elaine Kinsella, Anthony R. Leeds, John Norrie, Hemant G. Pandit, Hazel M. Ross, Sharon Anne Simpson, Chris Tuck

**Affiliations:** 1 Department of Trauma and Orthopaedics, University of Edinburgh, Chancellor’s Building, 49 Little France Crescent, Edinburgh, EH16 4SB UK; 2 Counterweight Ltd, 85 Great Portland Street, First Floor, London,, W1W 7LT UK; 3 Leeds Institute of Rheumatic and Musculoskeletal Medicine and NIHR Leeds Biomedical Research Centre, University of Leeds, 2nd Floor, Chapel Allerton Hospital, Chapeltown Road, Leeds, LS7 4SA UK; 4 MRC/CSO Social and Public Health Sciences Unit, University of Glasgow, 200 Renfield Street, Glasgow, G2 3QB UK; 5 Edinburgh Clinical Trials Unit, University of Edinburgh, Usher Institute, Level 2, Nine Edinburgh BioQuarter, 9 Little France Road, Edinburgh, EH16 4UX UK; 6 The Parker (arthritis) Institute, Copenhagen University Hospital, Bispebjerg og Frederiksberg, Nordre Fasanvej 57, DK-2000 Frederiksberg, Denmark

## Abstract

**Background:**

Patients’ pre-operative health and physical function is known to influence their post-operative outcomes. In patients with knee osteoarthritis, pharmacological and non-pharmacological options are often not optimised prior to joint replacement. This results in some patients undergoing surgery when they are not as fit as they could be. The aim of this study is to assess the feasibility and acceptability of a pre-operative package of non-operative care versus standard care prior to joint replacement.

**Methods/design:**

This is a multicentre, randomised controlled feasibility trial of patients undergoing primary total knee replacement for osteoarthritis. Sixty patients will be recruited and randomised (2:1) to intervention or standard care arms. Data will be collected at baseline (before the start of the intervention), around the end of the intervention period and a minimum of 90 days after the planned date of surgery. Adherence will be reviewed each week during the intervention period (by telephone or in person). Participants will be randomised to a pre-operative package of non-operative care or standard care. The non-operative care will consist of (1) a weight-loss programme, (2) a set of exercises, (3) provision of advice on analgesia use and (4) provision of insoles. The intervention will be started as soon as possible after patients have been added to the waiting list for joint replacement surgery to take advantage of the incentive for behavioural change that this will create. The primary outcomes of this study are feasibility outcomes which will indicate whether the intervention and study protocol is feasible and acceptable and whether a full-scale effectiveness trial is warranted.

The following will be measured and used to inform study feasibility: rate of recruitment, rate of retention at 90-day follow-up review after planned surgery date, and adherence to the intervention estimated through review questionnaires and weight change (for those receiving the weight-loss aspect of intervention). In addition the following information will be assessed qualitatively: analysis of qualitative interviews exploring acceptability, feasibility, adherence and possible barriers to implementing the intervention, and acceptability of the different outcome measures.

**Discussion:**

The aims of the study specifically relate to testing the feasibility and acceptability of the proposed effectiveness trial intervention and the feasibility of the trial methods.

This study forms the important first step in developing and assessing whether the intervention has the potential to be assessed in a future fully powered effectiveness trial. The findings will also be used to refine the design of the effectiveness trial.

**Trial registration:**

ISRCTN registry, ID: ISRCTN96684272. Registered on 18 April 2018.

## Background

The lifetime risk of knee osteoarthritis (OA) is 45% [1]. OA is the fastest growing cause of disability worldwide [2] and predicted to be the fourth leading cause of disability by 2020. Non-operative treatments, such as exercise, orthoses, analgesics and weight loss, are known to benefit patients with OA [3]. Patients with more severe OA are referred to secondary care for consideration of joint replacement surgery. Around 85,000 total knee arthroplasties (TKA) are performed annually in the UK [4]. Dissatisfaction with outcome is reported in around 20% of these cases [5, 6].

Patients’ pre-operative health and physical function is known to influence their post-operative outcomes. In patients with knee OA, pharmacological and non-pharmacological options are often not optimised prior to joint replacement. This results in some patients undergoing surgery when they are not as fit as they could be.

The overall aim of this project is to develop a complex intervention and an implementable package of care for patients listed for total knee replacement with the aims of improving post-operative outcomes and reducing complication risks. The aims of the study specifically relate to testing the feasibility and acceptability of the proposed effectiveness trial intervention and the feasibility of the trial methods.

This study aims to take advantage of the incentive for behavioural change in patients with osteoarthritis (OA) who have been placed on the waiting list for orthopaedic surgery, to obtain a durable alteration in the patients’ weight and exercise level. Non-operative treatments, such as exercise, orthoses, analgesics and weight loss, are known to benefit patients with OA [3] but are often not optimised prior to joint replacement. We postulate that the reduction in weight and increased activity coupled with an appropriate analgesia review and attention to footwear in the pre-operative window will result in a sustained improvement in the patient’s health-related quality of life following knee replacement.

### Exercise

The amount of exercise that osteoarthritic patients attending orthopaedic clinics carry out is very variable, yet there is good evidence that exercise (such as walking, swimming and cycling) reduces pain and improves physical function in patients with knee OA [7, 8]. The patients in this study are at the stage of listing for knee replacement and for them (unlike the early OA group for whom OARSI guidelines have been developed) the most efficacious exercise is muscle strengthening as this can enhance functional ability such as climbing stairs.

### Footwear

Shoes which have a thick shock-absorbing sole are considered most suitable for patients with lower-limb OA. In particular, there is evidence that in patients with knee OA, the use of shoes with shock-absorbing insoles for 1 month reduces pain and improves physical function [9].

### Analgesics

Many patients are not keen on taking strong painkillers, and compliance with OA pharmacological treatment is around 50% [10]. However, for mild to moderate pain, paracetamol can be effective. In addition, there is evidence that topical non-steroidal anti-inflammatory drugs (NSAIDs) may help relieve pain in knee and hand OA, yet many patients have not tried a range of simple analgesics.

### Weight loss

Obesity and being overweight are known to impair mobility and activity [11, 12]. Patients frequently have not lost weight prior to being listed for surgery and often have actually gained weight. There is evidence that for those who have knee OA being overweight can make symptoms worse for those who have knee OA [13].

In primary care, the effectiveness for patients with knee OA of weight-loss regimens on body weight, pain and/or physical function has been demonstrated in programmes delivered as weekly supervised sessions for a range of 8 weeks to 2 years [14–19]. In secondary care, in a study in New Zealand, where the waiting list times typically exceed 1 year, patients with a Body Mass Index (BMI) of over 40 were seen by a dietician. Surgery was withheld until the BMI was less than 40. Fifty-eight percent of patients lost weight [20]. A further study carried out in Denmark on osteoarthritic patients has demonstrated that a significant weight loss can occur in 8 weeks [21] and that a greater initial weight loss improves long-term maintenance of the reduction in weight.

#### Behavioural change

The package of care has a behavioural component which draws on Social Cognitive Theory, the Health Belief Model and Control Theory. It incorporates a number of evidence-based behaviour-change strategies to help people to change their behaviour initially and maintain this in the longer term, particularly in relation to the four elements of the intervention; adherence to medication, use of insoles, diet and physical activity. The key behaviour-change strategies include goal-setting, planning, self-monitoring and dealing with relapse. The intervention will also address key motivational aspects. Behavioural changes are often difficult to achieve in primary care [22]. However, a recent cluster-randomised trial [23] has demonstrated that smoking-cessation therapy given during a hospital in-patient episode had a much higher success rate suggesting that capitalising on the secondary-care episode could be an ideal time to change patients’ behaviour; especially if it is linked to the impending surgery.

The aim of the current research is to take advantage of this incentive for behaviour change in patients with OA attending orthopaedic clinics so that there is a durable change in the patients’ weight and exercise level, which, coupled with appropriate analgesia and insoles, could relieve the morbidity of their OA and improve outcomes following surgery. Recent evidence suggests that behaviour-change interventions may improve patient compliance with these non-surgical interventions and thus their success [23]. By optimising the non-operative treatment prior to surgery it may be possible to improve outcomes, such as knee pain and function, following surgery, reduce post-operative complications and at the same time improve the health-related quality of life of the patients.

Even though the proposed package of care of non-surgical interventions is recommended for this population, the delivery of, and adherence to, these interventions is variable. As recommended by the Medical Research Council guidance on the development and evaluation of complex interventions [24], we aim to carry out a feasibility study collecting both quantitative and qualitative data prior to embarking on a fully powered effectiveness randomised controlled trial (RCT). This study forms the important first step in developing and assessing whether the intervention has the potential to be assessed in a future fully powered effectiveness trial. The findings will also be used to refine the design of the effectiveness trial.

## Methods/design

### Objectives

The objective is to test the feasibility of the study methods and intervention delivery as well as the acceptability of the intervention in preparation for a fully powered effectiveness trial.

We aim to evaluate this by:

1. Investigating recruitment and retention
2. Investigating the feasibility and acceptability of the intervention (including assessment of the feasibility and acceptability of the intervention across a range of BMI values)
3. Exploring the barriers and facilitators to implementing the intervention
4. Assessing the feasibility and acceptability of different outcome measures
5. Estimating the variability in the key outcome measures, to inform the sample size calculations for the effectiveness trial
6. Estimating key cost-drivers to inform the design of a future economic evaluation

### Endpoints and process measures

#### Primary endpoint

The primary endpoint is whether the intervention and study protocol is feasible and acceptable and whether a full-scale effectiveness trial is warranted.

The following will be measured and used to inform study feasibility:

1. Rate of recruitment
2. Rate of retention at follow-up review after planned surgery date
3. Adherence to the intervention estimated through review questionnaires and weight change (for those receiving the weight-loss aspect of the intervention)

In addition the following information will be assessed qualitatively:

1. Qualitative interviews (with participants, researchers and clinical staff) exploring acceptability, feasibility, adherence and possible barriers to implementing the intervention
2. Acceptability of the different outcome measures

In addition to the above, a process evaluation will be carried out to explore in detail the way in which the intervention operates to produce outcomes. The evaluation will be conducted based on Medical Research Council guidelines for process evaluations of complex interventions and will examine the following elements: (1) context; (2) fidelity of the intervention; (3) exposure to the intervention; (4) reach; (5) recruitment and retention; (6) contamination; (7) the control arm; and (8) and mechanisms of impact. A logic model will be developed and tested as part of the process evaluation. A protocol for process evaluation will be prepared as a separate document.

#### Secondary endpoints

In the feasibility study we will monitor change for a minimum of 90 days after the planned surgery date in the outcomes we are interested in evaluating in the effectiveness trial. These are:

Change in:

1. Western Ontario and McMaster Universities Osteoarthriis Index (WOMAC) sub-scores
2. Change in Oxford Knee Score (OKS), EuroQol five dimensions questionnaire (EQ-5D) score, timed get-up-and-go test and self-efficacy questionnaire scores
3. Clinical outcomes following surgery (including routine complications)
4. Management of patient comorbidities
5. Patient satisfaction with knee pain and function
6. Identification of key cost drivers

## Study design

The study is a multicentre, randomised controlled feasibility trial which aims to assess the feasibility of running a pivotal randomised controlled trial (RCT) of a pre-operative package of non-operative care versus standard care prior to joint replacement.

We aim to recruit 60 participants (split between Edinburgh and Leeds sites). Recruitment is planned to take place over a 6-month period with a follow-up conducted for a minimum of 90 days after the planned surgery date.

Patients recently added to the waiting list for knee replacement surgery due to osteoarthritis will be approached about the study by a member of the clinical team at the outpatient clinic visit where the decision to proceed to knee surgery has taken place.

All participants will be seen for a baseline visit, where they will be randomised. Depending on the result of randomisation, patients will be asked to follow standard care or the intervention until their planned date of surgery (duration of intervention will depend on when knee surgery is due to take place). All participants will be seen in clinic at the end of the intervention period for a final review (pre-surgery) and a minimum of 90 days after the planned date of surgery. Participants in the intervention arm will also be reviewed regularly for the duration of the intervention to assess adherence.

A process evaluation (as described in ‘Section 2’) will be conducted.

## Study population

### Number of participants

#### Feasibility trial

Sixty participants will be recruited across two sites (roughly 30 in Edinburgh and 30 in Leeds) and randomized (2:1) to the intervention or standard care. It is important that both sites are involved in the feasibility study to establish that the design can fit with different service delivery models, to determine the generalisability of the intervention and the potential to expand to a multicentre effectiveness study.

Participant recruitment is planned to take place over a 6-month period and follow-up will be conducted a minimum of 90 days after the planned date of knee replacement surgery.

Participants will be stratified to ensure that we address the aim of testing the feasibility of recruiting people with a range of BMI values.

#### Process evaluation

Patients will be sampled purposively across a range of ages and trial participation status (refused to participate, agreed and retained to follow-up, agreed but did not complete the intervention) and approached to participate in the qualitative interviews. It is anticipated that up to 30 interviews will be required (split between Edinburgh and Leeds); data collection will continue until a point of saturation has been reached. All health care practitioners involved in the delivery of the intervention will be interviewed as well as up to five staff at each of the sites where the intervention is being delivered, including members of the participants clinical care team. Interviews will be conducted face to face and also by telephone according to the preference of the interviewee. Participants will also be invited to provide consent to allow recording of study reviews (where the research nurse is happy to do so) which will be analysed as part of the process evaluation.

### Inclusion criteria

For participants:

1. Undergoing a knee arthroplasty for OA
2. Participant meets at least one of the following threshold criteria:
  - BMI ≥ 30 kg/m^2^
  - Inability to perform straight-leg raise (no extensor lag) or patient-reported ‘giving way’
  - Not taking an appropriate level of analgesia
  - Not using shock-absorbing footwear
3. Participants are able to consent and willing to comply with the study protocol
4. Sufficient time for the intervention to be delivered before the planned date of surgery and for the follow-up appointment to be conducted after the planned date of surgery
5. Aged 18–85 years (inclusive)

For staff:

1. Staff members to be interviewed should be members of the research team involved in delivering the intervention to participants or members of the clinical care team/associated personnel at the site where the intervention is being delivered

### Exclusion criteria

For participants:

1. Patients undergoing revision knee arthroplasty or fully constrained knee arthroplasty
2. Knee replacement for a diagnosis other than OA
3. Patients with a second contralateral procedure planned within the study timeframe
4. Procedures done purely for pain relief (such as for patients with no walking capacity)
5. Patients involved in another research study containing elements of behaviour change related to diet, physical activity and other study elements
6. Patients currently under active review with a clinician for physiotherapy
7. Participants who cannot understand verbal explanations or written information given in English
8. Pregnant until > 4 months post-partum; breastfeeding

Additional exclusion criteria applicable to participants eligible for the weight-loss aspect of the intervention:

1. Patients who have recently lost a significant amount of weight (> 5 kg in the preceding 3 months) or who are already on a specialised diet
2. Patients with:
  - Insulin-dependent diabetes
  - Brittle type-2 diabetes which is managed in secondary care (confirmed by recent glycated haemoglobin (HbA1c) measurement if available)
  - Patients with moderate or severe retinopathy
3. Patients taking four or more anti-hypertensive agents
4. Patients with active mental illness: severe depression, bipolar disorders, schizophrenia or other psychotic disorders
5. Myocardial infarctions or stroke within the previous 3 months
6. Heart failure of grade III New York Heart Association or more severe
7. Porphyria
8. Substance abuse, e.g. drugs, alcohol
9. Eating disorder accompanied by purging (through laxative abuse or induced vomiting)
10. Previous bariatric surgery or scheduled bariatric surgery
11. Angina, arrhythmia, including atrial fibrillation or prolonged QT syndrome
12. Taking monoamine-oxidase inhibitor (MAOI) medication
13. Taking anticoagulant medication (e.g. warfarin)
14. Taking varenicline (smoking-cessation medication)
15. Chronic renal failure of stage 4 or 5 (as indicated by a recent estimated glomerular filtration rate (eGFR) reading of < 30 ml/min/1.73m^2)^
16. Patients:
  - With active liver disease (except non-alcoholic fatty liver disease (NAFLD)
  - With a history of hepatoma
  - Within 6 months of onset of acute hepatitis
17. People having active treatment for cancer other than skin cancer treated with curative intent by local treatment only, or people taking hormonal or other long-term secondary prevention treatment after initial cancer treatment
18. Active treatment or investigation for possible or confirmed gastric or duodenal ulcer; maintenance treatment with acid suppression is not a contraindication
19. Displaying symptoms associated with gallstones in the last 3 months
20. Not taking a proton-pump inhibitor if taking orally administered ibuprofen

Patients with type-2 diabetes being managed by their GP can be included if they are taking orally administered agents only or are diet-controlled type-2 diabetics.

Patients who have not responded to previous conservative management will not be excluded, indeed these patients are particularly interesting to see why their previous management failed, as this may have been due to a compliance problem.

Patients should be wiling to undertake the aspects of the package that they ‘qualify’ for based on the inclusion thresholds. Patients who trip several of the inclusion thresholds but meet the exclusion criteria for one or more will not be included.

For staff:

There are no exclusion criteria for staff.

### Co-enrolment

Co-enrolment will be permitted with other studies provided that the burden is acceptable to the participants and the study intervention does not involve any elements of behaviour change related to diet, physical activity and other study elements. Researchers must take care to not over-burden participants with requests to take part in research studies. Co-enrolment can only occur with studies where agreement has been obtained from the Chief Investigators (CIs) for each study.

## Participant selection and enrolment

### Identifying participants

Recruitment will be from the outpatient clinic where the decision to proceed to total knee replacement is made. A member of the clinical team will highlight the trial to the patient and, if interested in hearing more, the patient will be invited to complete a ‘Consent to Researcher Contact’ form and be provided with a patient information sheet. If it is known that a patient has type-2 diabetes and/or hypertension and appears to be eligible for the weight-loss aspect of the intervention, the ‘GP consent’ section of this form should also be completed (this will allow the research team to contact the patient’s GP in advance of randomisation to obtain consent regarding potential medication changes should the patient be randomised to the intervention arm). The patient will be given sufficient time to consider the study information, typically at least 24 h; however, contact may be made before this with the agreement of the patient. A member of the research team will discuss the study further with the patient and assess patient eligibility. Posters will also be placed in orthopaedic outpatient clinic areas for patients to highlight the study to site staff. Consent for the research team to contact the patient regarding the study may also be documented in a clinic letter from the consultant.

All staff delivering the intervention at sites will be invited to take part in an interview. A selection of staff involved in the care of individuals being seen at the outpatient clinic (including, but not limited to, orthopaedic surgeons doing knee replacement surgery, outpatient clinical staff dealing with knee replacement patients, managers involved in care delivery) at the centres taking part in the study will be invited to take part in an interview. These staff will be approached by members of the research team and provided with an information sheet and invited to complete a Consent to Contact Form. A sampling frame will be used which will include ensuring spread across the Leeds and Edinburgh sites and a range of relevant specialties and job roles.

### Consenting participants

If a patient agrees to take part in the study, a study visit will be arranged. Patient eligibility will be confirmed and written informed consent taken by a suitably qualified member of the research team.

Patients who are not interested in taking part in the study will be asked to consider taking part in an interview with the qualitative researcher only. Those that agree and would prefer the interview to be carried out over the telephone will be asked to provide their consent orally at the time of interview which will be audio-recorded. Participants who prefer to conduct the interview in person will be asked to provide written informed consent.

Clinical care team staff/associated personnel and the health care practitioners delivering the intervention who agree to be interviewed as part of the study will also be asked to provide either audio-recorded consent or written informed consent depending on their preference for interview.

#### Withdrawal of study participants

Participants are free to withdraw from the study at any point or a participant can be withdrawn by the Investigator. If withdrawal occurs, the primary reason for withdrawal will be documented on the patient’s Case Report Form (CRF). If participants choose to have their data removed from the analysis, this will be indicated on the appropriate form.

Withdrawal from study treatment will be distinguished from withdrawal from the study. Participants may choose to discontinue their participation in aspects of the intervention or study but remain active on the study (for example, to participate in the interview). The aspects of the study that the participant is happy to continue taking part in will be documented.

### Screening for eligibility

Participants will be identified and approached about the study by a member of the clinical team at the outpatient clinic where the decision to proceed to total knee replacement is made and then be formally screened by a member of the research team. Members of the clinical care team (or research team members, where it is locally agreed that they are part of the clinical care team, under the direct supervision of the site’s Principal Investigator (PI)) will review identifiable information (e.g. medical notes and clinic lists) to identify potential participants. They will not review or screen identifiable data that is outside normal clinical practice.

Participant eligibility may be verified by the research nurse. Confirmation of eligibility will be recorded within the participants’ medical records.

### Ineligible and non-recruited participants

Patients who are approached about the study but are found to be ineligible or refuse to take part will be recorded on the screening log. Patients who are not interested in taking part in the study will be asked to consider taking part in an interview with the qualitative researcher only. The reason for refusal, if provided, or ineligibility will be recorded on the screening log.

### Randomisation

#### Randomisation procedures

The allocation ratio is 2:1 between the Intervention and Standard Care treatment groups. Patients will be randomised at the baseline visit. Following randomisation, both the participant and the Investigator will be notified of the assigned treatment allocation.

A stratified list will be used to randomise the participants to a treatment group. Participants will be stratified by site and BMI band. BMI bands of < 30, ≥ 30 to < 35 and ≥ 35 will be employed. The stratified list will use block sizes of 3 and 6. The blocks will be randomly generated. The following additional constraints will be applied:

- At least one third of the participants recruited will have a BMI in the range ‘≥ 30 and < 35’
- At least one third of the participants recruited will have a BMI in the range ‘≥ 35’

Randomisation will be performed via a secure online randomisation system, set up by the Edinburgh Clinical Trials Unit, ensuring allocation concealment, and will be undertaken by an appropriate delegated member of trial team at study sites. In the event that the online randomisation process cannot be accessed there is a telephone back-up system available through the Edinburgh Clical Trials Unit (ECTU) (during office hours), details of which will be provided to the site teams.

#### Treatment allocation

Where a participant is randomised to the Intervention treatment group, they will be allocated one or more of the following aspects of the intervention. The aspects they qualify for will depend on the threshold criteria met.

- Analgesia
- Physical activity
- Weight-loss programme
- Insoles

## Study arms

Eligible patients will be randomised (2:1) to either the intervention or control arm. The duration of the intervention will be determined based on when the patient is due for surgery.

### Intervention arm

A logic model detailing the programme theory and an intervention manual for the study will be produced as separate documents.

Patients randomised to the intervention arm will be provided with the relevant elements of the package they ‘qualify’ for (listed below) by the research nurse delivering the intervention at the baseline visit.

#### Behaviour change

This is a fundamental ingredient of the intervention which underlies the other aspects listed below. These behaviour-change techniques utilised as part of this intervention in relation to these different elements are; goal-setting, self-monitoring, providing feedback, planning, barrier identification, problem-solving, engaging social support and dealing with relapse.

#### Analgesia review

A member of the research team will review the use and timing of appropriate topical and orally administered analgesics with the patient. If the participant meets the inclusion criteria related to analgesia usage, a member of the research team will discuss advice in line with routine clinical care regarding painkillers that may help with pain related to knee osteoarthritis.

#### Physical activity

Participants who are unable to perform a straight-leg raise (extensor lag) or report ‘giving way’ will be provided with exercises to do at home. The exercise package will be tailored to the individual patient based on their pain symptoms experienced during physical activity [25]. Strengthening exercise of the lower limb will be targeted to facilitate enhanced functional activity with daily tasks. The focal intervention will be quadriceps muscle strengthening through a graded progression of load and intensity (to target giving way/straight-leg-raise lag symptoms). Specific parameters surrounding individual exercise selection, frequency, load and intensity will vary with the patient’s ability and baseline strength [26]. Exercises will be performed at home without the need for equipment. Additionally, the patient’s current levels of physical activity will be reviewed and aerobic exercise will be promoted as pain allows.

#### Weight-loss programme

A weight management programme comprising a Low Energy Liquid Diet (LELD) will be offered to all whose BMI is > 30 kg/m^2^. The programme will utilise the Counterweight-Plus programme and will comprise a Total Diet Replacement (TDR) phase of 800 kcal/day for 8–12 weeks. For those participants receiving the weight-loss intervention enough diet resource will be provided for approximately 2 weeks and then replenished at the fortnightly review visits with the local research team. The Counterweight-Plus programme includes a LELD and is an effective intervention [27, 28]. It is manualised to maintain the fidelity of the intervention. Participants offered the LELD will be given structured advice for the period of the intervention. After the TDR phase, advice and information on weight-loss maintenance and food reintroduction will be provided. This aims to increase the sustainability of the TDR, maximising the likelihood that weight loss will be maintained after the 8–12 week TDR phase [27, 28]. The local research team will discuss the plan for food reintroduction with the participant (this will be individualised based on the time available before surgery) and will provide support around phased food reintroduction to allow them to return to a normal diet. To facilitate this, a minimum period of at least 1 week should be left between the end of the intervention and the date of surgery. Depending on the time available before surgery, some patients may progress to the Weight Loss Maintenance phase. Advice on weight-loss maintenance and support will be provided by the research team.

#### Insoles

If patients are not already routinely using shock-absorbing insoles or are deemed not to routinely wear shoes with shock-absorbing soles, they will be provided with shock-absorbing insoles. This component of the intervention is supported by NICE and EULAR guidelines and will help minimise limitation from OA. It is considered that minimising the restriction caused by OA will help patients comply with physiotherapy and weight reduction.

#### Delivery of the intervention

The intervention will be delivered by members of the research team who have been trained accordingly. Participants will attend for a baseline assessment and provision of the specific package of conservative interventions including the behaviour-change component. Face-to-face review sessions will take place at fortnightly intervals to deliver relevant intervention components (including the behaviour-change component) and to assess adherence to the intervention and to re-supply the dietary resource. Apart from the clinic-based reviews, the intervention will be ‘home’ based for the intervention period. On the weeks in between visits for the first 8–12 weeks, a member of the research team at each centre will make weekly review telephone calls to the participant to deliver relevant intervention components (including the behaviour-change component) and to assess adherence to the intervention.

#### Participant compliance

Compliance with the randomisation allocation will be assessed using data collected through review questionnaires and weight change (for those receiving the weight-loss aspect of the intervention) for the purposes of the study outcomes. Non-compliance with the randomised allocation will not be recorded as a protocol deviation and/or violation.

#### Medication changes related to the weight-loss programme

Participants with type-2 diabetes (being managed by their GP) and/or hypertension who are randomised to the intervention arm of the study and qualify for the weight-loss aspect of the study may find that the doses of drugs they need to control their diabetes and high blood pressure need to be reviewed. Research staff may discuss potential medication changes with participants and GPs (with participants’ consent). Further information and advice for research staff will be provided in the study intervention manual and the medication management plan. Decisions regarding medication changes are down to the individual participant’s situation and GP preference. A medication management plan (which has been formulated from previous studies using the Cambridge Weight Plan) will be agreed with these participants’ GP before the intervention is started and will be employed by site PIs (and sub-investigators delegated to perform this task) when changes to medication are required. Site PIs (and/or sub-investigators delegated to perform this task) will be responsible for decisions made regarding medication changes. GPs will be informed of medication changes made by the PI. Hypertensive participants’ blood pressure and diabetic participants’ blood glucose levels will be monitored at clinic visits. In addition to the schedule outlined in ‘Section 7’, diabetic participants with home blood glucose monitors may be advised to monitor their blood glucose levels at home as required.

### Control arm

Participants in the control arm will receive usual local standard care prior to total knee replacement at their local treatment centre. Care received and any change in the trial parameters (weight loss, analgesia usage, etc.) in the control arm will be documented and evaluated to determine any behaviour change in this arm brought about through the informed consent process or completion of study questionnaires.

## Study assessments

An example summary of study assessments and visits is provided in Table 1 and a schematic of the patient journey in Additional file 1 Figure S1.

**Table 1:** Example study schedule for a patient receiving all aspects of the intervention with 17 weeks between randomisation and planned surgery date

Visit	Pre-op															Post-op
	ortho-paedic clinic visit	Baseline visit	Phone review	Clinic review	Phone review	Clinic review	Phone review	Clinic review	Phone review	Clinic visit^3^	Phone review	Clinic review	Phone review	Final review (clinic)	Surgery	follow-up clinic visit
		Week														
		0	1	2	3	4	5	6	7	8–12	13	14	15	16	17	
All participants:																
Consent to researcher contact	X															
PIS	X															
Inclusion/exclusion criteria		X														
Informed consent		X														
Demographic details		X														
Medical history		X														
Height		X														
Weight		X	X^2^	X		X		X		X		X		X		X
Blood pressure		X												X		X
Questionnaires^1^		X												X		X
Pain score		X	X^2^	X		X		X		X		X		X		X
Timed get-up-and-go test		X												X		X
Randomisation		X														
Adherence review			X^2^	X	X	X	X	X	X	X	X	X	X	X		
Interviews^4^			X													X
Satisfaction questionnaire																X
Intervention arm only:																
Intervention delivery		X	X^2^	X	X	X	X	X	X	X	X	X	X	X		
Weight-loss aspect only:																
ESE questionnaire		X												X		X
BEQ/EDE questionnaires		X								X ^6^				X ^6^		X ^6^
Blood pressure			X^2^	X		X		X		X		X		X		X
Blood glucose		X	X^2^	X		X		X		X		X		X		X
Total Diet Replacement phase			X	X	X	X	X	X	X	X						
FR/WLM phase^5^											X	X	X	X		

^1^Questionnaires include Western Ontario and McMaster Universities Osteoarthritis Index (WOMAC), EuroQol five dimensions questionnaire (EQ-5D), OKS, pain self-efficacy scale, arthritis self-efficacy and eating self-efficacy scale (for those participants receiving the weight loss aspect of the intervention)

^2^Additional clinic visit for participants with diabetes and/or hypertension only who are receiving the weight loss aspect of the intervention. An additional blood pressure measurement will be collected for participants with hypertension. An additional blood glucose measurement for those participants with type-2 diabetes will be collected. A weight measurement will also be collected at this visit and a clinic review Case Report Form (CRF) completed

^3^The Total Diet Replacement (TDR) will be delivered for a minimum of 8 weeks and a maximum of 12 weeks. The final visit of the TDR phase should be a clinic visit to allow collection of weight and blood pressure measurements and delivery fo advice on food reintroduction

^4^Interviews will be conducted at an appropriate time before surgery or during the 90-day follow-up period

^5^*FR* Food Reintroduction, *WLM* Weight Loss Maintenance. Depending on the time available before surgery, some patients may progress to the Weigh Loss Maintenance phase

^6^Binge-eating questionnaires should be repeated at the end of TDR, end of FR and at follow-up if a binge-eating disorder was identified at the baseline visit and if participants display issues with eating food while on TDR

Potential participants will be assessed against the inclusion and exclusion criteria as much as possible over the telephone by a member of the research team to determine eligibility. If deemed potentially eligible, participants will be invited to attend for a baseline visit.

### Baseline visit (all participants)

Participants will be asked to provide their consent to take part in the study. Participants will be assessed against the inclusion and exclusion criteria to confirm eligibility. Participants who are eligible for the weight-loss aspect of the study will be asked to complete a binge-eating scale questionnaire and eating-disorder examination questionnaire to assess the presence of an eating disorder. Participants eligible for the weight-loss aspect of the intervention will be invited to try a sample of the weight-loss product before randomisation. Basic demographic details (including height, weight and blood pressure measurements) and medical history will be collected from all participants. A timed-get-up-and-go test will be performed; this is the time that a person takes to rise from a chair, walk 3 m, turn around, walk back to the chair, and sit down and will be measured. The following questionnaires will also be completed: WOMAC, OKS, EQ-5D, arthritis self-efficacy, pain self-efficacy scale and a pain questionnaire. These questionnaires are those proposed for use in the effectiveness trial. Participants will then be randomised.

Participants randomised to the control arm will receive usual local standard care prior to total knee replacement for the period between the start of intervention and their planned date of surgery.

Participants randomised to the intervention arm will be provided with whichever elements of the package they ‘qualify’ for by a member of the research team. Participants will receive the intervention for the period between the start of intervention and their planned date of surgery. All services/interventions received in the control group will be recorded.

Participants who are to receive the weight-loss aspect of the intervention will be asked to complete an eating self-efficacy scale questionnaire.

Participants with diabetes receiving the weight-loss aspect of the intervention will have blood glucose levels checked at this visit using a blood glucose meter.

### Telephone reviews (participants in the intervention arm)

Participants will be contacted by telephone every 2 weeks (± 3 days) by a member of the local research team and a review assessing the participant’s general health and adherence to the intervention will be completed. Relevant intervention components (including the behavioural component will also be delivered. These calls will occur approximately every 2 weeks (starting on week 1) after initiating the intervention until the end of the TDR phase for participants on the weight-loss programme or for 8–12 weeks for participants receiving the other aspects of the intervention (depending on the time available before surgery).

For participants with diabetes and/or hypertension, the week-1 review telephone call will be a clinic visit in order to measure blood pressure and blood glucose levels.

### Clinic reviews (participants in the intervention arm)

Relevant intervention components (including the behavioural component) will be delivered in person every 2 weeks (± 3 days) by a member of the local research team and a review assessing the participants’ general health and adherence to the intervention will be completed. Participants will be asked to complete a pain questionnaire. These visits will occur approximately every 2 weeks (starting on week 2) after initiating the intervention until the end of the intervention period. After the end of the TDR phase for participants on the weight-loss programme or after 8–12 weeks for participants receiving the other aspects of the intervention (depending on the time available before surgery), participants will be encouraged to come to clinic to complete these reviews; however, they may be conducted by telephone if preferred.

At these clinic reviews, weight will be collected from those participants receiving the weight-loss aspect of the intervention. Blood pressure measurements will be collected from all participants. Participants with diabetes who are receiving the weight-loss aspect of the intervention will have blood glucose levels checked at these visits.

For participants with diabetes and/or hypertension, the week-1 review telephone call will be a clinic visit in order to measure blood pressure and blood glucose levels.

A clinic review visit should be carried out at the end of the TDR phase for those participants undertaking the weight-loss programme where there is more than 1 week available for food reintroduction. At this visit, advice and information on food reintroduction will be provided. The local research team will discuss the plan for food reintroduction with the participant and support will be provided around phased food reintroduction to allow the participant to return to a normal diet before surgery. A sample food reintroduction plan will be prepared as a separate document; however, for each patient the plan will be individualised based on the time available before surgery. A period of at least 1 week should be left between the end of the TDR and the date of surgery to facilitate the food reintroduction process.

Weight-loss programme participants will be asked to complete a binge-eating scale questionnaire and an eating-disorder examination questionnaire at this end of TDR and end of Food Reintroduction phases if a binge-eating disorder was identified at the baseline visit and if participants display issues with eating food while on TDR.

Depending on the time available before surgery, some patients may progress to the Weight Loss Maintenance phase. Patients who progress to this phase will be provided with appropriate advice on weight-loss maintenance and supported by the study team until their planned surgery date.

### Final review (all participants)

Participants will complete a final study review at the hospital 1 week (± 3 days) before the planned surgery date. Patients in the control arm may have their Final Review clinic visit aligned with their pre-admission clinic visit where the pre-admission clinic visit is no more than 3 weeks before surgery. All services/interventions received in the control group will be recorded.

Participants will complete a review assessing their general health and adherence to the intervention. A timed-get-up-and-go test will be performed in addition to the following questionnaires: WOMAC, OKS, EQ-5D, arthritis self-efficacy, pain self-efficacy scale, a pain questionnaire and an eating self-efficacy scale (for those participants receiving the weight-loss aspect of the intervention). Weight and blood pressure measurements will be collected from all participants. Participants who have undertaken the weight-loss aspect of the study will be asked to complete a binge-eating scale questionnaire and an eating-disorder examination questionnaire at the end of the Food Reintroduction phase if a binge-eating disorder was identified at the baseline visit and if participants display issues with eating food while on TDR. Participants with diabetes receiving the weight-loss aspect of the intervention will have blood glucose levels checked at this visit. A period of at least 1 week should be left between the end of the intervention and the date of surgery to facilitate the food reintroduction process.

Participants on the weight-loss programme will be provided with information on weight-loss maintenance if not already received.

All services/interventions received in the control group will be recorded as part of the process evaluation.

### Follow-up review (all participants)

A review will be completed in clinic with participants a minimum of 90 days after the planned date of surgery. A timed-get-up-and-go test will be performed and participants will be asked to complete the following questionnaires: WOMAC, OKS, EQ-5D, arthritis self-efficacy, pain self-efficacy scale, a pain questionnaire and an eating self-efficacy scale (for those participants receiving the weight-loss aspect of the intervention). Weight and blood pressure measurements will be collected from all participants. Participants who have undertaken the weight-loss aspect of the study will be asked to complete a binge-eating scale questionnaire and an eating-disorder examination questionnaire if a binge-eating disorder was identified at the baseline visit and if participants display issues with eating food while on TDR. Participants with diabetes that were receiving the weight-loss aspect of the intervention will have blood glucose levels checked at this visit.

Participants will also be asked to complete a satisfaction questionnaire at this time point. Data on complications experienced 90 days after surgery will be collected at this visit and from the participants’ medical notes.

### Process evaluation

All participants (control and intervention arm) will be invited to participate in an interview at the time of consent. Participants will also be invited to provide consent to allow recording of study reviews (where the research nurse is happy to do so) which will be analysed as part of the process evaluation. Interviews will explore participants’ attitudes towards the study processes and feasibility and acceptability of the intervention as well as assessing barriers and adherence issues. Participants who refuse to take part in the interview or refuse recording of study reviews will still be eligible to take part in the main study. Qualitative interviews will be conducted with up to 30 participants, sampled purposively across a range of ages and trial participation status (refused to participate, agreed and retained to follow-up, agreed but did not complete the intervention). Interviews with control and intervention participants will be conducted at an appropriate time before surgery or during the 90-day follow-up period. Interviews with individuals who declined participation or withdrew from the study will be conducted at an appropriate time during the trial period. All participant reviews will be audio-recorded (where consent has been provided to do so).

Health care practitioners involved in the delivery of the intervention will be invited to be interviewed as well as up to five staff at each of the sites where the intervention is being delivered. These interviews will be conducted at an appropriate time during the trial period and will be conducted face to face or by telephone according to the preference of the interviewee. The qualitative interviews will be facilitated by a qualitative researcher with input from the qualitative lead on the study (Professor Sharon Simpson).

## Data collection and storage

See Table 1 for an example schedule of study assessments and time points. Trial data will be collected on paper data collection sheets and transcribed into the project database, a web-based data entry system, at site by the research team. Data will be collected in clinic or over the telephone by a member of the research team. Personal data and study data collected on paper forms will be stored in a locked filing cabinet in a secure location at the recruitment centre.

Data regarding complications following surgery (e.g. deep vein thrombosis (DVT), infections, etc.) will be collected from the hospital records throughout the participants’ participation in the study to rehearse the data collection methods for the effectiveness trial. Participants will also be asked about any problems during the intervention period reviews, final review (pre-surgery) and 90-day follow-up visits.

Qualitative data will be collected by audio-recording and/or on paper forms during study reviews and interviews with a subset of participants, health care practitioners involved in delivering the intervention and staff at the sites where the intervention is being delivered. Audio data will be recorded using encrypted digital recording devices (with minimum encryption standard of AES256 equivalent) and downloaded to password-protected folders on networked drives at the University of Glasgow. Recordings will then be deleted off the recording device. Data will be subsequently transcribed (a third party may be employed to transcribe the data) after which recordings will be deleted. The qualitative researcher employed to carry out the process evaluation will be responsible for the secure storage of data.

The trial database will be stored on secure servers at the University of Edinburgh. This database will have restricted, username- and password-controlled access. A Data Management Plan will be prepared that will include the following details as a minimum: CRF development and management; CRF workflow, data-entry processes and location of source data; training and user access; data quality; quality control; and database lock. Additional details regarding the management of study data may be added to the Data Management Plan where applicable. Designated staff at ECTU will follow ECTU Standard Operating Procedures (SOPs) to obtain missing data and resolve queries with site staff and to ensure data quality and completeness of data across sites. A Data Quality Check Plan will be prepared as a separate document.

## Statistics and data analysis

### Sample size calculation

#### Randomised trial (feasibility)

This is a feasibility study and so its aim is to assess the acceptability and practicality of delivering the intervention, and to estimate parameters for a larger study. As part of the feasibility design, we plan a 6-month recruitment window to estimate the participation rate for the future effectiveness trial. In terms of sample size, the choice of 60 is appropriate to see if a meaningful recruitment rate can be achieved. This size of feasibility study has been recommended by Lancaster et al. [29]. In terms of looking more formally at feasibility, a sample of 60 randomised participants would allow us to estimate proportions such as the proportion compliant with the different aspects of the intervention or the proportion completing the trial.

As most participants (estimated to be over 80% from our pilot data) will require multiple components of the intervention, the retention outcome relates to the overall package. Retention within the trial, applicable to all 60 randomised subjects, will be estimated with a standard error (SE) of approximately 6%. Compliance with the package of care, applicable to the 40 participants randomised to the intervention group, will be estimated with a SE of approximately 8%.

Participants will be stratified to ensure that we address the aim of testing the feasibility of recruiting people with a range of BMI values.

#### Qualitative interviews

Qualitative interviews will be conducted with approximately 30 patients sampled purposively across a range of ages and trial participation statuses, research staff involved in the delivery of the intervention and up to five staff at each of the sites where the intervention is being delivered.

### Proposed analyses

As this is a feasibility study the quantitative data will be presented descriptively, using appropriate summary statistics with corresponding 95% confidence intervals. Additionally, the results will be summarised separately for the two sites, and no formal comparisons will be made. The key outcome measure is the number of participants recruited per centre over the 6-month recruitment period, which will be supported by the preparation of a Consolidated Standards of Reporting Trials (CONSORT) flow chart per centre showing the ‘pipeline leakage’ from potentially eligible patients listed for surgery through to eligible, consented, randomised patients.

Rates of compliance with each relevant component of the intervention will also be reported along with completion rates for all outcome measures. The feasibility trial is not powered to explore efficacy, but the outcome data will be used to estimate the standard deviation (SD) of the data for the different outcome scales (especially WOMAC), and the impact on this SD of baseline adjustment. These estimates of variability will be used to inform the sample size calculation for the effectiveness trial. There are no plans to conduct interim analyses while recruitment is on-going or before follow-up is completed.

In designing this study we have followed the MRC guidance for developing and evaluating complex interventions [24]. This guidance indicates that rigid progression criteria are not appropriate. In collaboration with the Trial Steering Committee (TSC) we shall develop a straightforward decision model that will identify key barriers and enablers of recruitment, retention and compliance, and use this to decide whether and how we can adapt the design to overcome any problems we encounter.

#### Qualitative analyses

Qualitative data will be analysed using Braun and Clarks’ approach to thematic analysis [30]. The resulting coding framework will be discussed within the team to finalise meaningful themes and sub-themes. Twenty percent of the interviews will be double coded. Disagreements will be resolved by discussion. The analyses will test the hypothesised causal pathways expressed in the logic model. This will inform the study design of any future trial by determining which elements of the intervention work well for health-behaviour change in participants, how they interact with each other and which need adjustment or further development.

The audio-recordings of sessions will be analysed using a checklist developed as part of the process evaluation. The researchers will listen to the audio-recordings and code absence or presence of different core parts of the intervention for the purposes of checking intervention fidelity.

#### Key cost-driver analysis

Identification of key cost drivers will be done through consultations with local experts to discuss the types of NHS services expected to be major and minor drivers differences in costs in patients undertaking the intervention or standard care. This will include consultation with the lead health economist (Andrew Stoddart), working on the analysis of NHS costs of the TRIO physio trial (a trial of physiotherapy for patients with poorly performing knee transplants, CI: Hamish Simpson). This trial included a baseline self-reported NHS resource survey at 6 weeks post surgery profiling service use in the preceding 3 months. These results will be visually inspected to identify factors which appear to constitute most substantially to total cost figures in order to inform future survey design. Any other transferable lessons from the trial will also be documented.

## Additional information

### Oversight and safety reporting

The study is sponsored by the Academic and Clinical Central Office for Research and Development – Joint Office for The University of Edinburgh and Lothian Health Board (ACCORD) and relevant SOPs have been applied to this study. Safety reporting will be conducted in line with relevant sponsor SOPs. Study-specific particulars are detailed in the complete trial protocol. A Trial Management Group and a TSC has been established.

### Confidentiality and data protection

All evaluation forms, reports, and other records must be identified in a manner designed to maintain participant confidentiality. All records must be kept in a secure storage area with limited access. Clinical information will not be released without the written permission of the participant. The Investigator and study site staff involved with this study may not disclose or use for any purpose other than performance of the study, any data, record, or other unpublished, confidential information disclosed to those individuals for the purpose of the study. Prior written agreement from the sponsor or its designee must be obtained for the disclosure of any said confidential information to other parties.

All Investigators and study site staff involved with this study must comply with the requirements of the Data Protection Act 1998 with regard to the collection, storage, processing and disclosure of personal information and will uphold the Act’s core principles. Access to collated participant data will be restricted to individuals from the research team treating the participants, representatives of the sponsor(s) and representatives of regulatory authorities.

Computers used to collate the data will have limited access measures via user names and passwords.

Published results will not contain any personal data that could allow identification of individual participants.

### Authorship policy and publication

Ownership of the data arising from this study resides with the Trial Management Group. On completion of the study, the study data will be analysed and tabulated and a clinical study report prepared in accordance with International Conference on Harmonisation (ICH) guidelines. A separate publication policy will be prepared for the study.

The clinical study report will be used for publication and presentation at scientific meetings. Summaries of results will be made available to Investigators for dissemination within their clinics (where appropriate and according to their discretion).

## Discussion

The aims of the study specifically relate to testing the feasibility and acceptability of the proposed effectiveness trial intervention and the feasibility of the trial methods.

We will recruit patients recently added to the waiting list for knee replacement surgery, aiming to take advantage of the incentive for behaviour change in these patients. Patients will be randomised to receive a package of care which incorporates behaviour-change strategies to help people to change their behaviour initially and maintain this in the longer term, particularly in relation to the four elements of the intervention. We postulate that a reduction in weight and increased activity coupled with an appropriate analgesia review and attention to footwear in the pre-operative window will result in a sustained improvement in the patient’s health-related quality of life following knee replacement.

This study forms the important first step in developing and assessing whether the intervention has potential to be assessed in a future fully powered effectiveness trial. The findings will also be used to refine the design of the effectiveness trial.

## Supplementary information

**Additional file 1.** Standard Protocol Items: Recommendations for Interventional Trials (SPIRIT) 2013 Checklist: recommended items to address in a clinical trial protocol and related documents.

## Data Availability

Not applicable

## Abbreviations

ACCORD: Academic and Clinical Central Office for Research and Development – Joint Office for The University of Edinburgh and Lothian Health Board
AE: Adverse Event
AR: Adverse Reaction
ARUK: Arthritis Research UK
BMI: Body Mass Index
CI: Chief Investigator
CRF: Case Report Form
EQ-5D: EuroQol five dimensions questionnaire
GCP: Good Clinical Practice
ICH: International Conference on Harmonisation
ISF: Investigator Site File
NSAIDS: Non-steroidal anti-inflammatory drugs
OA: Osteoarthritis
OARSI: The Osteoarthritis Research Society International
OKS: Oxford Knee Score
PI: Principal Investigator
PISCF: Patient Information Sheet and Consent Form
QA: Quality Assurance
R&D: Research and Development
REC: Research Ethics Committee
SAE: Serious Adverse Event
SAR: Serious Adverse Reaction
SOP: Standard Operating Procedure
TDR: Total Diet Replacement
TSC: Trial Steering Committee
WOMAC: Western Ontario and McMaster Universities Osteoarthritis Index
