# Correction to: Osteoarthritis Preoperative Package for care of Orthotics, Rehabilitation, Topical and oral agent Usage and Nutrition to Improve ouTcomes at a Year (OPPORTUNITY); a feasibility study protocol for a randomised controlled trial

**DOI:** 10.1186/s13063-020-04292-4

**Published:** 2020-04-20

**Authors:** A. Hamish R. W. Simpson, Colin R. Howie, Elaine Kinsella, David F. Hamilton, Philip G. Conaghan, Catherine Hankey, Sharon Anne Simpson, Anna Bell-Higgs, Peter Craig, Nicholas D. Clement, Catriona Keerie, Sarah R. Kingsbury, Anthony R. Leeds, Hazel M. Ross, Hemant G. Pandit, Chris Tuck, John Norrie

**Affiliations:** 1 Department of Trauma and Orthopaedics, University of Edinburgh, Chancellor’s Building, 49 Little France Crescent, Edinburgh, EH16 4SB UK; 2 Edinburgh Clinical Trials Unit, University of Edinburgh, Usher Institute, Level 2, Nine Edinburgh BioQuarter, 9 Little France Road, Edinburgh, EH16 4UX UK; 3 Leeds Institute of Rheumatic and Musculoskeletal Medicine and NIHR Leeds Biomedical Research Centre, University of Leeds, 2nd Floor, Chapel Allerton Hospital, Chapeltown Road, Leeds, LS7 4SA UK; 4 MRC/CSO Social and Public Health Sciences Unit, University of Glasgow, 200 Renfield Street, Glasgow, G2 3QB UK; 5 Counterweight Ltd, 85 Great Portland Street, First Floor, London, W1W 7LT UK; 6 The Parker (arthritis) Institute, Copenhagen University Hospital, Bispebjerg og Frederiksberg, Nordre Fasanvej 57, DK-2000 Frederiksberg, Denmark

**Correction to: Trials**

**https://doi.org/10.1186/s13063-019-3709-5**

After publication of our article [1] we have been notified that two of the author names have been mistakenly removed from the authorship list: Colin R. Howie and Nicholas D. Clement.

The order of the authors has also been corrected.

Initially published authorship:

A. Hamish R. W. Simpson1*, Anna Bell-Higgs2†, Philip G. Conaghan3†, Peter Craig4†, David F. Hamilton1†, Catherine Hankey4†, Catriona Keerie5, Sarah R. Kingsbury3†, Elaine Kinsella5, Anthony R. Leeds6†, John Norrie5†, Hemant G. Pandit3†, Hazel M. Ross2†, Sharon Anne Simpson4† and Chris Tuck5†.
